# Characteristics and outcomes of people living with HIV hospitalised at tertiary healthcare institutions during the COVID-19 pandemic in Mexico City

**DOI:** 10.1186/s12879-024-09208-0

**Published:** 2024-05-24

**Authors:** Yanink Caro-Vega, Lorena Guerrero-Torres, Andrea Cárdenas-Ortega, Alexandra Martin-Onraët, Patricia Rodríguez-Zulueta, Karla Romero-Mora, María Gómez-Palacio Schjetnan, Alicia Piñeirúa-Menéndez

**Affiliations:** 1 Departamento de Infectología, Instituto Nacional de Ciencias Médicas y Nutrición Salvador Zubirán, Mexico City, Mexico; 2 Departamento de Infectología, Instituto Nacional de Enfermedades Respiratorias, Mexico City, Mexico; 3 Departamento de Infectología, Instituto Nacional de Cancerología, Mexico City, Mexico; 4 Departamento de Infectología, Hospital General Dr. Manuel Gea González, Mexico City, Mexico; 5 CISIDAT, Cuernavaca, Morelos México; 6 Dwight Morrow, 8-7, Cuernavaca Centro, Cuernavaca Morelos, 62000 Mexico

**Keywords:** SARS-CoV-2, AIDS, Hospitalisation, México, Latin America, Observational

## Abstract

**Background:**

While existing research on people living with HIV (PWH) during the COVID-19 pandemic primarily focused on their clinical outcomes, a critical gap remains in understanding the implications of COVID-19 delivery of in-hospital care services to PWH. Our study aimed to describe the characteristics and outcomes of PWH hospitalised during 2020 in Mexico City, comparing patients admitted due to COVID-19 vs. patients admitted due to other causes.

**Methods:**

All PWH hospitalised for ≥ 24 h at four institutions in Mexico City from January 1st to December 31st, 2020 were included. Patients were classified into two groups according to the leading cause of their first hospitalisation: COVID-19 or non-COVID-19. Characteristics among groups were compared using chi-square and Kruskal tests. A Cox model was used to describe the risk of death after hospitalisation and the characteristics associated with this outcome. Mortality and hospitalisation events were compared to data from 2019.

**Results:**

Overall, we included 238 PWH hospitalised in 2020. Among them, 42 (18%) were hospitalised due to COVID-19 and 196 (82%) due to non-COVID-19 causes, mainly AIDS-defining events (ADE). PWH hospitalised due to COVID-19 had higher CD4 + cell counts (380 cells/mm3 [IQR: 184–580] vs. 97 cells/mm3 [IQR: 34–272], *p* < 0.01) and a higher proportion of virologic suppression (VS) compared to those hospitalised due to non-COVID-19 causes (92% vs. 55%, *p* < 0.01). The adjusted hazard ratio (aHR) for AIDS was 3.1 (95%CI: 1.3–7.2). COVID-19 was not associated with death (aHR 0.9 [95%CI: 0.3–2.9]). Compared to 2019, mortality was significantly higher in 2020 (19% vs. 9%, *p* < 0.01), while hospitalisations decreased by 57%.

**Conclusions:**

PWH with COVID-19 had higher VS and CD4 + cell counts and lower mortality compared to those hospitalised due to non-COVID-19-related causes, who more often were recently diagnosed with HIV and had ADEs. Most hospitalisations and deaths in 2020 in PWH were related to advanced HIV disease. The increased mortality and decreased hospitalisations of PWH during 2020 evidence the impact of the interruption of health services delivery for PWH with advanced disease due to the pandemic. Our findings highlight the challenges faced by PWH during 2020 in a country where advanced HIV remains a concern.

## Background

The devastating toll of the COVID-19 pandemic is evident in the 3.0 million deaths due to COVID-19 in the Region of the Americas since the onset of the pandemic [1]. Beyond the direct impact of SARS-CoV-2 infection, the disruption of healthcare services for unrelated causes resulted in excess mortality [1–6]. Among the groups affected, people living with HIV (PWH) faced significant challenges in accessing essential HIV services due to COVID-19 prevention measures worldwide [7, 8]. Studies have reported a decline in new HIV diagnoses, reduced in-hospital HIV testing rates, discontinuation of mental health care services, disruption of sexually transmitted infections (STIs) and vaccination programs, and an increase in individuals diagnosed with advanced disease (defined as CD4 + cell count < 200 cells/mm^3^) [7–10].

In Mexico City, the leading ambulatory clinic specializing in HIV care recorded a 33% reduction in HIV testing during 2020 compared to the previous year, coupled with a 10% increase in the proportion of PWH with advanced disease at diagnosis [11]. The repercussions of COVID-19 on HIV programs threaten to achieve the 95-95-95 targets set for 2030, especially in nations such as Mexico, where a substantial 43% of PWH are still diagnosed with advanced disease [12–14]. Adding to the complexity, the tertiary care institutions in Mexico, historically responsible for managing PWH with advanced disease, prioritized care of people with SARS-CoV-2 infection, mirroring the global trend experienced in 2020 [15].

While existing research primarily focused on the clinical outcomes of PWH with COVID-19 and HIV [16–21], a critical gap remains in understanding the implications of COVID-19 delivery of in-hospital care services to PWH [22]. Our study aimed to address this knowledge gap by describing the characteristics and outcomes of PWH hospitalised during the COVID-19 pandemic in Mexico City, comparing those hospitalised due to COVID-19 infection to those hospitalised due to other causes.

## Methods

### Study Population

The study included all PWH admitted at four institutions in Mexico City: Instituto Nacional de Cancerología (INCan), Instituto Nacional de Ciencias Médicas y Nutrición Salvador Zubirán (INCMNSZ), Instituto Nacional de Enfermedades Respiratorias (INER), and Hospital General Dr. Manuel Gea González (HGMGG); for a duration of 24 h or more between January 1st to December 31st, 2020. PWH were identified through administrative discharge databases of the institutions mentioned above, ensuring that all eligible individuals were accounted for in the study. These institutions are tertiary care centres affiliated with the Ministry of Health (MoH) and provide care to uninsured patients from Mexico City and other central states. Notably, all four centres have an ambulatory HIV clinic and possess the capacity to provide hospital care to PWH when necessary. It is important to highlight that these centres transitioned to functioning exclusively as COVID-19 centres, adhering to federal guidelines and directing their in-hospital resources solely towards individuals with COVID-19 starting from mid-March 2020 [15]. As per the protocol in the four institutions, a SARS-CoV-2 real-time reverse transcription-polymerase chain reaction (RT-PCR) test was performed on all patients with clinical symptoms of COVID-19 and all asymptomatic patients admitted for other reasons (i.e. chemotherapy, surgery) upon admission. We followed all included individuals for 180 days after their last discharge.

### Procedures

This was a retrospective study. We designed a database to gather information from medical records systematically. We developed a codebook to ensure consistency and standardisation, establishing precise definitions for each variable. We reviewed the medical records of PWH to ascertain admission and discharge dates related to in-hospital events and to detail the characteristics and outcomes of these hospitalisations. We included all hospitalisation events per individual in the analysis, with outcomes derived from the final hospitalisation for PWH experiencing multiple admissions. We collected data on HIV infection (including viral load, CD4 + cell count and antiretroviral treatment) from +/-365 days of hospital admission. Although Mexican HIV Guidelines recommend semiannual viral load measurements and annual CD4 + cell count assessments for virologically suppressed [23], the COVID-19 pandemic prompted a shift in this approach. To mitigate healthcare service saturation and potential in-clinic COVID-19 transmission, regular follow-up services were temporarily spaced for stable individuals on antiretroviral therapy (ART) and achieving virological suppression. Consequently, most patients underwent only one viral load and CD4 + cell count assessment the preceding year.

All HIV diagnoses occurring after the date of admission to the initial hospitalisation event were considered in-hospital HIV diagnoses. Virological suppression (VS) was defined as a viral load below 200 copies/mL. We registered all hospitalisation causes and clinical outcomes within 180 days following the last hospitalisation. We included all deaths within 30 days after the final hospitalisation event discharge.

We classified patients into two groups based on the cause of their primary hospitalisation: COVID-19 or non-COVID-19. The COVID-19 group comprised individuals with confirmed SARS-CoV-2 infection based on a positive RT-PCR test or, in cases of negative RT-PCR, a high clinical suspicion supplemented by chest computed tomography (CT) findings indicative of COVID-19 pneumonia [24]. Conversely, the non-COVID-19 group comprised individuals with negative SARS-CoV-2 RT-PCR results and a clinical course inconsistent with COVID-19. We defined advanced HIV disease by a CD4 + T cell count below 200 cells/mm^3^. We estimated the number and type of AIDS-defining events (ADE) from the full spectrum of diagnoses recorded in hospital charts for each hospitalisation. Clinical hospitalisation outcomes (including discharge due to clinical improvement, discharge for end-of-life care at home, referrals, and in-hospital deaths) were documented, focusing on the last hospitalisation event. Mortality and the main causes of death were recorded from the clinical charts.

As a countermeasure to the scarcity of beds for PWH not afflicted by COVID-19 during the transformation of tertiary care hospitals into exclusive COVID-19 centres, a unique situation emerged from April to August 2020. Some non-COVID-19 PWH requiring hospitalisation were referred to a private hospital for treatment. This arrangement, facilitated by an agreement between the MoH and a private healthcare hospital, entailed the financial burden being shouldered by the MoH and a private foundation. Physicians from the HIV clinics in the converted hospitals were assigned to clinical duties to provide care to these patients. This cohort is integrated into the results collectively with the rest of the sample. The Research and Ethics Committee of Instituto Nacional de Ciencias Médicas y Nutrición Salvador Zubirán reviewed and approved this study on May 17th, 2021, with reference number 3748. Due to the retrospective nature of the study, considering that the information was obtained from the files, and given that no specific samples were taken from the participants, the Research and Ethics Committee of Instituto Nacional de Ciencias Médicas y Nutrición Salvador Zubirán waived the Informed Consent.

### Statistical analyses

We calculated frequencies and proportions of categorical variables of all study individuals stratified according to COVID-19 and non-COVID-19 groups. We estimated the median and interquartile range of continuous variables (age, duration from HIV diagnosis to first hospitalisation admission, CD4 + cell count during hospitalisation, and in-hospital days during the initial stay). We compared group characteristics through chi-square and Kruskal-Wallis tests.

We used a Cox model to describe the risk of death after hospitalisation and the characteristics associated with this outcome. The observation period commenced from the admission date of the initial hospitalisation and concluded at death (within 30 days post the final hospitalisation event discharge) or the latest available date: discharge of the last event or the last clinical visit within the next six months. The model included variables such as sex, age, COVID-19 group, and the interaction between AIDS diagnosis and HIV diagnosis during hospitalisation and was stratified based on the institution of hospitalisation. Age was modelled as a continuous variable, employing splines with three nodes. We built the adjusted survival curve from this Cox model and reported the number of at-risk patients at each point. We plotted the predicted adjusted survival from the Cox model by COVID-19 group and AIDS status.

We described the number of hospitalisation events, hospitalised individuals, mean in-hospital duration (in days), and the proportion of deaths among PWH during 2019 in the same institutions. Using the Chi-square test, we compared the percentages of patients with multiple events and the percentage of deaths between 2019 and 2020. We conducted all statistical analyses using R version 3.6.1.

## Results

### Study Population

We analysed data from 238 PWH hospitalised at the participating institutions during the specified timeframe. From those 30 PWH were hospitalised in the private health care center. The participants had a median age of 38 years (interquartile range [IQR]: 30–49), of whom 10% (24/238) were female. Among the participants, 54% (128/238) had an ADE during their initial hospitalisation. 12% (28/238) received their HIV diagnosis during their first hospitalisation, a proportion that increased to 39% (92/238) when considering both the first hospitalisation and the preceding six months. For those with over six months of HIV diagnosis, the median duration from HIV diagnosis to the first hospitalisation was 8.1 years (IQR: 3.6–14.6).

Among 90% (214/238) of PWH, a CD4 + cell count was available, measured at a median of 29 (IQR: 9–78) days before the first hospitalisation event. The participants had a median CD4 + cell count of 125 cells/mm^3^ (IQR: 44–341). Furthermore, 87% (207/238) of PWH had a viral load measurement from a median of 32 days (IQR: 3–84) before their first hospitalisation. Among those with available viral load data, 62% (129/207) had VS.

Of the participants, 18% (42/238) were hospitalised due to COVID-19, while 82% (196/238) were due to non-COVID-19 causes. All participants hospitalized in the private center were classified in the non-COVID-19 group. Among those with COVID-19 diagnosis, 95% (40/42) had a positive RT-PCR test for SARS-CoV-2, while 5% (2/42) were supported by characteristic pulmonary CT image and a clinical trajectory strongly suggestive of COVID-19 pneumonia. Since oxygen support was the main requirement for hospitalization according to the local guidelines at the time, all PWH hospitalized due to COVID-19 required oxygen support due to acute respiratory failure. However, specific data regarding oxygen saturation at hospital admission was not collected for all patients [25]. A comparative analysis of patient characteristics for each group is detailed in Table 1.

**Table 1:** Characteristics of patients with HIV hospitalised for COVID and non-COVID causes in 2020

	All *N* = 238 (100%)	COVID-19 *n* = 42 (18%)	Non-COVID-19 *n* = 196 (82%)	p-value
Median age, years (IQR)	38 (30–49)	46 (40–54)	36 (30–45)	< 0.001
Sex, women (%)	24 (10)	2 (5)	22 (11)	0.268
Median time since HIV diagnosis, months (IQR)^†^	26 (1–114)	136 (43–236)	9 (1–93)	< 0.001
In hospital HIV diagnosis, number (%)	28 (12)	1 (2)	27 (14)	0.061
Median CD4 count during hospitalisation, cells/mm^3^ (IQR)^†^	125 (44–341)	380 (184–580)	97 (34–272)	< 0.001
Undetectable viral load during hospitalisation (VL < 200), number (%)^†^	129 (62)	35 (92)	94 (55)	< 0.001
AIDS-defining events during hospitalisation, number (%)	128 (54)	3 (7)	125 (64)	< 0.001
Median days of in-hospital stay (IQR)	14 (7–25)	13 (5–18)	15 (7–27)	0.187
Clinical outcomes of hospitalisation, number (%)^‡^ Discharge due to improvement Discharge for end-of-life care at home Referral Death	179 (75) 2 (< 1) 14 (6) 43 (18)	35 (83) 0 (0) 2 (5) 5 (12)	144 (73) 2 (1) 12 (6) 38 (19)	0.586
Two or more hospitalisations, number (%)^*^	40 (17)	1 (2)	39 (20)	0.003

^†^ Includes only first hospital admission and patients with viral load available *N* = 207, 37 in the COVID and 170 in the non-COVID-19 group

^‡^ The last hospitalisation outcomes are described for patients with more than one hospitalisation.

IQR = Interquartile range.

Patients in the non-COVID-19 group were younger and had a lower median CD4 + cell count during hospitalisation, a higher proportion of ADEs, and a lower proportion of VS upon admission than PWH in the COVID-19 group.

### Hospitalisation events

We identified a total of 300 hospitalisation events. Predominantly, 83% (198/238) of PWH experienced a single hospitalisation event during 2020. Those with multiple events had a median count of 2 (IQR: 2–3). PWH hospitalised due to non-COVID-19 causes demonstrated a higher proportion of two or more hospitalisations (20% vs. 2%, *p* < 0.01). A length of stay of at least 14 days during the initial hospitalisation was observed in 49% (116/238) of PWH. Considering all events, the median hospitalisation time for PWH was 14 days (IQR: 7–25), totalling 4,467 patient-days.

The primary reasons for first hospitalisation were ADEs (54%, 128/238), exclusive COVID-19 (16%, 39/238), and other causes (30%, 71/238). Among the identified ADEs, the most prevalent were Kaposi Sarcoma (KS) (26%, 33/128), *Pneumocystis jirovecii* pneumonia (23%, 29/128), tuberculosis (19%, 24/128), histoplasmosis (15%, 19/128) and non-Hodgkin Lymphoma (15%, 19/128). Among the PWH hospitalised due to COVID-19, three also had ADEs. All three had histoplasmosis, one had tuberculosis, and one had KS. Other causes of hospitalisation were non-ADE infections (61%, 43/71), elective surgeries (13%, 9/71), systemic diseases (13%, 9/71), non-ADE cancer or chemotherapy administration (8%, 6/71), urgent surgeries (3%, 2/71), mental health disorders (1%, 1/71) and pregnancy-related conditions (1%, 1/71).

### Outcomes

The most common clinical outcome was discharge due to improvement, constituting 75% (179/238) of cases. Overall mortality was 18% (43/238), with 12% (5/42) of PWH in the COVID-19 group and 19% (38/196) in the non-COVID-19 (*p* = 0.36). In the non-COVID-19 group, 66% (25/38) of deaths were attributed to ADEs, while 24% (9/38) were attributed to non-ADE infections or malignancies, and 10% (4/38) were due to other causes. In this group, 5 deaths were recorded among the participants hospitalised in the private center. Among the PWH with COVID-19, 43% (18/42) required invasive mechanical ventilation (IMV), of which 78% (14/18) were intubated. All deaths in the COVID-19 group were due to Acute Respiratory Failure Syndrome, with four of them occurring during IMV. Additionally, two further deaths were observed in the non-COVID-19 group, occurring at 61 and 107 days after the final hospitalisation event. The first death resulted from an ADE, while the second was attributed to other causes.

**Fig. 1:**
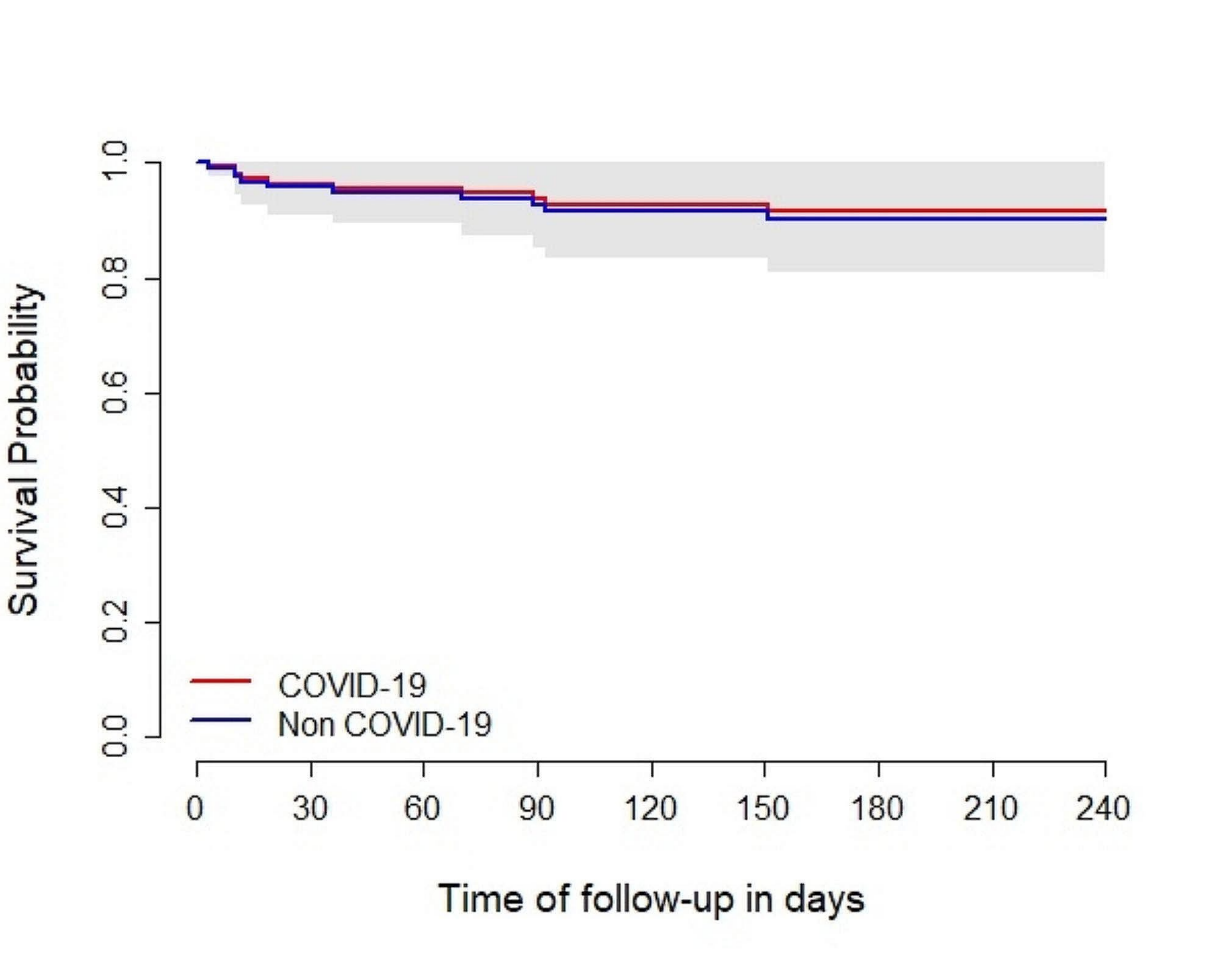
Adjusted survival probability since the first hospitalisation for COVID-19 vs. non-COVID-19. *Note* Adjusted survival from a Cox model for death, stratified by COVID group, including sex, age, COVID group, and the interaction between AIDS diagnosis and HIV diagnosis during hospitalisation. The curves are predicted survival curves using male sex, 40 years old, non-HIV diagnosis and non-AIDS status. The grey area depicts the 95% confidence interval

**Fig. 2:**
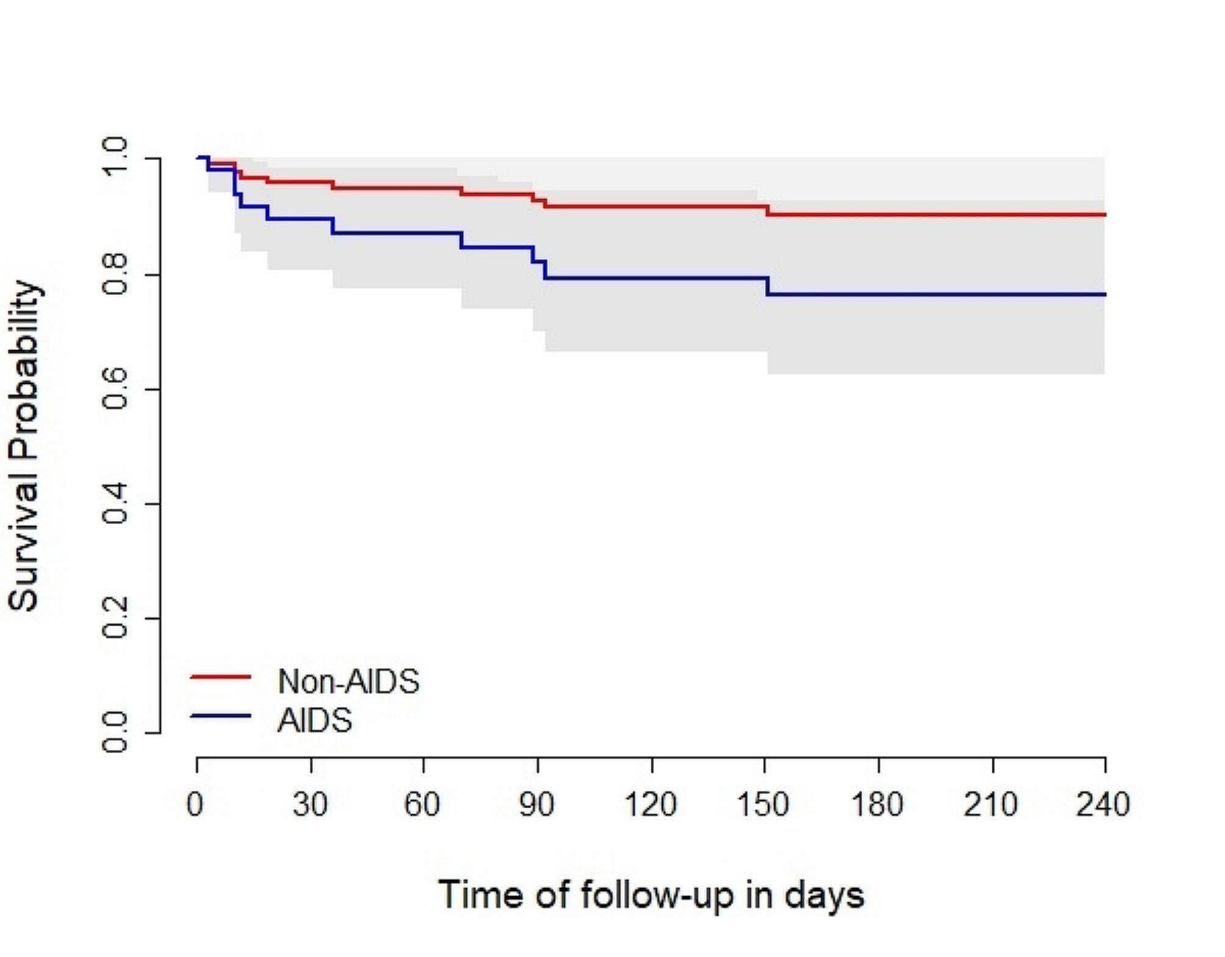
Adjusted survival probability since the first hospitalisation for AIDS vs. non-AIDS. *Note* Adjusted survival from a Cox model for death, stratified by COVID group, including sex, age, COVID group, and the interaction between AIDS diagnosis and HIV diagnosis during hospitalisation. The curves are predicted survival curves using male sex, 40 years old, non-HIV diagnosis during hospitalisation, and no-COVID status. The grey area depicts the 95% confidence interval.

In a Cox model examining mortality, an adjusted Hazard Ratio (aHR) of 2.63 (95% CI: 1.14–6.08, *p* = 0.023) was associated with AIDS diagnosis. As an admission diagnosis, COVID-19 was not associated with death, yielding an aHR of 0.86 (95% CI: 0.28–2.62, *p* = 0.79) compared to other admission causes (Figs. 1 and 2). Additional factors associated with mortality among hospitalised PWH are outlined in Table 2.

**Table 2:** Factors associated with death among hospitalised PWH

	aHR	95% CI	p-value
COVID-19 vs. no-COVID-19	0.86	0.28–2.61	0.793
AIDS vs. non-AIDS	2.62	1.13–6.08	0.023
HIV diagnosis during hospitalisation	0.69	0.08–5.64	0.731
Age at hospitalisation 30 40 50 60	1.28 1.38 1 0.60	0.73–2.24 1.00–1.92 1 0.36–1.02	0.186
Female vs. Male	1.16	0.43–3.10	0.767

Note: The adjusted hazard ratios (aHR) were estimated from a Cox model for death during hospitalisation, including the institution as a stratum and the interaction between HIV diagnosis and AIDS condition (*p* = 0.9). CI = Confidence interval, PWH = People living with HIV.

### General characteristics of hospitalisation events during 2019

To assess the impact of COVID-19 on hospitalisation and mortality among PWH, we compared the characteristics of PWH hospitalised in the same centres during 2019 and those in 2020. In 2019, we documented 812 events involving 556 PWH (Table 3). The median in-hospital duration was ten days (IQR: 6–16.2) accumulating to 11,131 patient-days. Among the PWH, 25% (140/556) experienced multiple hospitalisations, and 9% (51/556) died.

In comparison to 2019, we documented a decline of 57% in the total number of PWH hospitalised (238 vs. 556), along with a 60% reduction in total patient-days (4,467 vs. 11,131 days). Although the proportion of PWH experiencing more than one hospitalisation event was lower (17% vs. 25%, *p* = 0.01), the mortality among all PWH was significantly higher (18% vs. 9%, *p* < 0.01) in 2020 compared to 2019. Moreover, within the non-COVID-19 group, the mortality in 2020 was significantly higher than that observed in 2019 (19% vs. 9%, *p* < 0.01).

**Table 3:** General characteristics of hospitalisation events in 2019 and 2020

	2019	2020	p-value
Number of events	812	300	NA
Number of PWH hospitalised	556	238	NA
Total patient-days	11,131	4467	NA
PWH with two or more hospitalisations, number (%)	140 (25)	40 (17)	0.012
Median days of in-hospital stay (IQR)	10 (6–16.2)	14 (7–25)	0.049
Number of deaths, number (%)	51 (9)	43 (18)	< 0.001

IQR = Interquartile range

## Discussion

This study thoroughly compares clinical characteristics and mortality risks between PWH hospitalised due to COVID-19 and non-COVID-19 causes throughout 2020 within four tertiary care centres in Mexico City. We highlight two distinctive populations: one encompassing PWH with COVID-19, characterised by CD4 + counts over 350 cells/mm^3^, a high prevalence (92%) of VS and a mortality rate of 12%. The other group consists of PWH with recent HIV diagnosis requiring hospitalisation due to ADEs and displaying a high AIDS-related mortality. Our study reports a substantial male representation (76%) among the participants, reflecting the sex distribution of PWH in the country and particularly in Mexico City. Mexico has a concentrated HIV epidemic, revealing a national male-to-female ratio of 5:1 and a ratio of 13:1 within Mexico City, where the study was conducted [26].

Prior research examining HIV and SARS-CoV-2 co-infection typically compares PWH outcomes to those without HIV. While some studies associate HIV with an increased hospitalization or death risks, often linked to factors like low CD4 cell counts and comorbidities [16–21, 27], our study found a 12% COVID-19 related mortality rate among PWH, consistent with the average reported across studies (16%) [28]. Notably, most PWH with COVID-19 in our study had CD4 + cell counts above 350 cells/mm^3^ and viral suppression, reinforcing that well-controlled HIV does not lead to higher mortality than that of the general population [28, 29].

Comparatively, few studies have examined COVID-19 versus non-COVID-19 hospitalisations in PWH [30]. Our study shows that PWH hospitalised due to non-COVID-19 causes had significantly lower CD4 + counts, a high proportion of ADEs and higher mortality (19%). A proportion of our patients (14%) received private care due to the transformation of specialized hospitals into COVID-19 centers. This underscores the critical need for uninterrupted specialised care, especially given the high rate (40%) of advanced HIV diagnosis among Mexican PWH [14].

The disruption of healthcare services during the pandemic has had a profound effect, leading to delayed HIV diagnosis and increased late diagnoses, interruptions in treatment, and reduced access to preventive services [31, 32]. Meanwhile, adaptability in outpatient services has shown resilience, with modifications to clinical follow-ups for PWH during the pandemic [33]. However, our study reveals the alarming impact of COVID-19 on in-patient services, showing a 60% reduction in patient-days and increased mortality (18% vs. 9%) in PWH who were hospitalised for non-COVID causes in 2020 compared to those hospitalized in 2019, highlighting the importance of maintaining in-hospital care services for vulnerable populations, especially during health emergencies. To mitigate future crises, strategies should focus on ensuring continuous specialized care for vulnerable populations like PWH; establish robust preparedness and response plans to ensure uninterrupted HIV service delivery, including access to prevention and treatment pathways, routine vaccinations and use of telehealth platforms to access care [34, 35]. Additionally, it is imperative to strengthen health information systems so that they can integrate data from different sources, including outpatient and inpatient settings to better monitor and respond to unanswered questions that contribute to improve public health guidelines on COVID-19 or other emergent infectious diseases and HIV.

Our study has some limitations due to its retrospective nature, potentially leading to biases. First, our hospitals lacked a standardised protocol for HIV testing among people admitted for COVID-19 care, potentially excluding those who were unaware of or did not disclose their HIV status [36]. This may limit the representativeness of the study’s findings and potentially affect the generalizability of the results. Additionally, we acknowledge recent research indicating the value and cost-effectiveness of concurrent HIV and SARS-CoV-2 testing protocols to diagnose early HIV infection, rapidly link to care and initiate antiretroviral treatment [37–39]. Second, although we analysed data from four hospitals that provide care to a considerable number of HIV patients in Mexico City, our data solely reflects the experiences within the capital and might not be broadly applicable to other Mexican regions. Furthermore, information on CD4 + cell counts and viral load for PWH hospitalised in 2019 was unavailable. Regardless of this gap in our data, we can infer from the increased mortality rates and the nature of hospital presentations that the pandemic likely exacerbated the vulnerability of PWH, potentially due to the interruptions in HIV care as reported globally and locally [31, 32, 40–42]. Nonetheless, we consider that in regions with limited access to specialised HIV care beyond Mexico City, outcomes for PWH with advanced disease could potentially been worse.

Despite these limitations, the strengths of our study lie in the data quality and rigorous analytical methods. Expert physicians reviewed clinical charts and classified hospitalisations and deaths. By comparing PWH with COVID-19 and non-COVID-19 and using multivariate methods, we could account for clinical differences, especially regarding the AIDS and COVID-19 status, and assess their association with the risk of death post-hospitalisation.

## Conclusions

In conclusion, PWH hospitalized due to COVID-19 were older, had higher VS and CD4 + cell counts, and had lower mortality compared to those hospitalised due to non-COVID-19 causes, who more often were recently diagnosed with HIV and had ADEs. Our study also reveals an important reduction in access to specialised tertiary healthcare for PWH with advanced disease in 2020, accompanied by a concerning surge in mortality attributable to non-COVID-19 causes compared to the previous year. This trend appears to be primarily driven by the disruption of specialised HIV care within tertiary hospitals in Mexico City. The findings underscore the challenges faced by PWH during the initial year of the COVID-19 pandemic in a country where advanced disease at diagnosis remains a concern. The data offers valuable insights to identify potential strategies and interventions to enhance timely access to care, particularly for PWH experiencing ADEs. Urgent efforts are required to build resilient health systems capable of effectively addressing the needs of vulnerable populations.

## Data Availability

The data that support the findings of this study are available from the corresponding author upon reasonable request.
